# A novel risk score model based on five angiogenesis-related long non-coding RNAs for bladder urothelial carcinoma

**DOI:** 10.1186/s12935-022-02575-1

**Published:** 2022-04-19

**Authors:** Xinyuan Li, Chunlin Zhang, Xiang Peng, Yang Li, Guo Chen, Xin Gou, Xiang Zhou, Chao Ma

**Affiliations:** 1 Department of Urology, The First Affiliated Hospital of Chongqing Medical University, No. 1 Youyi Road, Yuzhong District, Chongqing, 400016 China; 2 CAS Centre for Excellence in Molecular Cell Science, Shanghai Institute of Biochemistry and Cell Biology, Chinese Academy of Sciences, Shanghai, China; 3 The Fifth People’s Hospital of Chongqing, Chongqing, China; 4 Chongqing Key Laboratory of Molecular Oncology and Epigenetics, Chongqing, China

**Keywords:** Bladder urothelial carcinoma, Angiogenesis, Tumour microenvironment, Long noncoding RNA, Risk model, Prognosis

## Abstract

**Background:**

Tumour angiogenesis is an independent risk factor for bladder urothelial carcinoma (BUC) progression, but viable and promising antiangiogenic targets are understudied. Emerging evidence suggests that long non-coding RNAs (lncRNAs) play prominent role in the tumour microenvironment and tumour angiogenesis.

**Methods:**

The clinical data of BUC patients were obtained from TCGA database and clinical specimens of 138 BUC patients. Univariate and multivariate COX regression analyses were used to identify survival-related ARLNRs (sARLNRs) from The Molecular Signatures Database v4.0. Fisher’s exact probability method was used to detect the correlations between sARLNRs levels and clinicopathological characteristics. A chain of experiments including FACS, qPCR, immunohistochemistry, tube formation, migration and invasion assays, combining with co-culture models, were utilized to validate the clinical significance and angiogenetic correlation of sARLNRs.

**Results:**

Five sARLNRs were employed to establish an angiogenesis-related risk score model, by which patients in the low-risk group obtained better overall survival than those in the high-risk group. The expression of AC005625.1 and AC008760.1 was significantly related to ECs percentage, tumour size and muscle invasion status. Besides, AC005625.1 and AC008760.1 expressed lower in BUC cell lines and tumour tissues than that in normal urothelial cells and adjacent normal tissues, with much lower levels in more advanced T stages. A prominently higher proportion of ECs was detected in tumour tissues with lower expression of AC005625.1 and AC008760.1. In the co-culture models, we found that knockdown of AC005625.1 and AC008760.1 in BUC cells increased the tube formation, migration and invasion abilities of HUVEC. The expression levels of CD31, VEGF-A, VIMENTIN and N-CADHERIN were also enhanced in HUVEC cells co-cultured with siR-AC005625.1 and siR-AC008760.1-treated T24 cells.

**Conclusion:**

In the study, we identify five sARLNRs and validate their clinical significance, angiogenesis correlation and prognosis-predictive values in BUC. These findings may provide a new perspective and some promising antiangiogenic targets for clinical diagnosis and treatment strategies of BUC.

**Supplementary Information:**

The online version contains supplementary material available at 10.1186/s12935-022-02575-1.

## Introduction

Bladder urothelial carcinoma (BUC) is the most common genitourinary malignancy with 500,000 new cases and 200,000 deaths per year worldwide [1]. BUC can present as non-muscle-invasive bladder cancer (NMIBC) accounting for near 80% patients diagnosed with BUC, muscle-invasive bladder cancer (MIBC) with higher aggression or as a metastatic disease [2]. Despite the 5-year OS of NMIBC is greater than 85%, it has not significantly improved for a good while, and the frequent recurrence also put a heavy load on the quality of life (QOL) [2–4]. Representing the remaining 20% of localized disease, MIBC get the higher disease-specific mortality and the worse life expectancy, with approximately 50% 5-year OS [1].

Although surgery, chemotherapy and immunotherapy have been widely applied for BUC, few survival improvements have been achieved until an emerging body of next-generation sequencing help us better understand the genetic variations and molecular alterations, identify actionable therapeutic targets, and predict patient prognosis [5, 6]. Besides, the clear cellular geography and the acknowledge of various cell types allow us improve the awareness of genetic background better understand a considerable variety and heterogeneity of the tumour [7]. Recently, intense efforts are being made to clarify the genome‐wide association of BUC and identify more valuable candidate genes for diagnostic markers, and therapeutic targets [5]. The variants of aldehyde dehydrogenase (ALDH2) enzyme were demonstrated to be closely associated with the higher recurrence rate of BUC [8]. CD44 polymorphisms probably responding for the susceptibility of BUC were potential for a molecular prognostic marker [9]. Additionally, the identification of some mutated genes such as FGFR3 [10], TP53 [11] and ERCC2 [12, 13], led to a resurgent interest in the realm of targeted therapy. An emerging body of studies at the genetic level are discovering more attractive and viable targets.

Tumour angiogenesis, a highly dynamic and complex pathological process of new vessel formation in the primary site or metastatic site, has been identified as an independent prognostic indicator in many cancers [14, 15]. By supplying abundant nutrition and natural migration pathway, angiogenesis promotes tumour progression and regulates tumour microenvironment. Therefore, antiangiogenetic targeted therapy combined with anti-tumour cells approaches seems to be an alternative way for cancer therapy [16]. Additionally, identifying some promising angiogenetic markers has also been shown as a viable strategy for diagnosis and prognosis estimation, but is still in its infancy, especially for BUC [17, 18].

Long non-coding RNAs (lncRNAs), a group of non-coding RNAs that are more than 200 base pairs in length, are dynamically expressed in variety of biological activities including tumour angiogenesis [19, 20]. LncRNAs affect angiogenesis by regulating various angiogenetic molecules, endothelial cell behaviors and tube formation. Besides, a cascade of lncRNAs have been found to participate in the angiogenesis-related pathways, such as vascular endothelial growth factor (VEGF) [21, 22] and Notch [23]. Therefore, angiogenesis-related lncRNAs (ARlncR) as a kind of potential candidate, merit further discovery and attention in the field of antiangiogenetic strategies.

## Materials and methods

### Clinical sample collection

Tumour tissues and adjacent normal tissues of 138 BUC patients who underwent tumour excision or tissue biopsy in the First Affiliated Hospital of Chongqing Medical University between March 2019 and February 2021 were collected. (Table 1) Patients with severe underlying diseases or other primary cancers were excluded. The collected clinical tissues were utilized to analyze the expression of lncRNAs and percentage of endothelial cells (ECs). All patients included in this study provided written informed consent, and this study was approved by the Medical Ethics Committee of the First Affiliated Hospital of Chongqing Medical University (IRB:2021-085). All clinical data were reviewed according to medical records.

**Table 1:** Relationship between AC005625.1 and AC008760.1 expression and clinicopathologic factors of UBC patients

Parameter	N	Average expression of sARLNRs		*P* value
		Low	High	
Gender				0.361
Male	115	60	55	
Female	23	9	14	
Age (year)				0.305
< 65	75	34	41	
≥ 65	63	35	28	
ECs percentage (%)				**< 0.001**
< 25	69	18	51	
≥ 25	69	51	18	
Tumor size (cm)				**0.003**
< 2	58	20	38	
≥ 2	80	49	31	
Muscle invasion status				**0.022**
Negative	52	19	33	
Positive	86	50	36	
Lymph node status				0.208
Negative	132	64	68	
Positive	6	5	1	
Distant metastasis status				0.098
Negative	123	58	65	
Positive	15	11	4	
Nidus number				
Single	126	64	62	0.764
Multiple	12	5	7	

The bold number represents the *P*-values with significant differences

### Cell culture

SV-HUC-1, BUC cell lines (T24, UM-UC-3, 5637, J82 and TCC-SUP) and HUVEC were purchased from the American Type Culture Collection (Manassas, Virginia, USA). Cells were cultured in DMEM (SV-HUC-1, UM-UC-3, T24 and HUVEC), McCoy’s 5A (J82) and RPMI-1640 (5637 and TCC-SUP) basal medium (Gibco, Gaithersburg, MD, USA), which were supplemented with 10% fetal bovine serum (FBS, MilliporeSigma, Burlington, MA, USA), 100 U/ml penicillin and 0.1 mg/ml streptomycin (Beyotime, Beijing, China). Cells were incubated at 37 °C in 5% CO_2_ incubator. The medium was changed every 1–3 days.

### Tissue processing

Bladder specimens were obtained fresh from the operating field where grossly apparent tumour tissue or adjacent tissue not grossly affected by tumour (Par-cancer tissue). These tissues were transported at room temperature immersed in the RPMI-1640 medium (Gibco, Gaithersburg, MD, USA) with 10% FBS (MilliporeSigma, Burlington, MA, USA). Once received, tumour tissues were divided into two parts, one of which was cut into approximately 1 mm^3^ pieces and enzymatically digested to single cell suspensions using MACS tumor dissociation kit (Miltenyi Biotec) for 1 h on a rotor at 37 °C for further flow cytometry/FACS analysis, another part of tumour tissues and all par-cancer tissues were frozen in liquid nitrogen immediately and then stored at − 80 °C until lncRNA extraction.

### ECs percentage detection by flow cytometry/FACS

The single cell suspensions were filtered with screen cloth and cell surface staining was performed in FACS buffer containing CD31 antibody (Cat# 303102, BioLegend, USA) on ice for 1 h. Following washing twice with FACS buffer, the percentages of EC subtype (CD31 positive) in these single cell suspensions were detected using a FACS flow cytometry system (Cytoflex, Beckman Coulter, USA).

### Cell transfection

The siRNAs of AC005625.1 and AC008760.1 were used to silence the expression of AC005625.1 and AC008760.1. The sequences used were: si-AC005625.1 (sense: 5′-GCUUCACAGCCACCAUCUATT-3′, antisense: 5′-UAGAUGGUGGCUGUGAAGCT T-3′); si-AC008760.1 (sense: 5′-GACAGGUAGUCACGACUAUTT-3′, antisense: 5′-AUAGUC GUGACUACCUGUCTT-3′). For transient transfection, T24 cells were added into 6-well plate (1 × 10^5^ cells per well). When cells grew to 50–60% confluence, cells were transfected with 10 μl siRNAs (20 nM) using 5 μl Lipofectamine 3000 (Invitrogen, USA) for 48 h according to the manufacturer. Finally, we used RT-qPCR to evaluate the expression levels of lncRNAs.

### Real-time quantitative PCR

We used Trizol (Takara) to extract total lncRNAs from tissues and cell lines under various experimental conditions. cDNA Synthesis Kit (Takara) combined with lncRNAs (1 μg) was utilized to reverse transcribed cDNA. The quantitative polymerase chain reaction (qPCR) was performed on an ABI 7500 real-time PCR system (Applied Biosystems) by SYBR-Green method (Takara). The values of Ct were calculated with the 2^−ΔΔCt^ method and normalized to the expression levels of β-actin. The expression levels of lncRNAs were relative to the fold change of their controls which were defined as 1. The primer sequences were shown in Table 2. Three assays were conducted per cDNA sample.

**Table 2:** The primer sequences of AC005625.1, AC008760.1 and β-actin

AC005625.1	F primer (5′–3′)	TTGTTTGTTGTTCGCCACC
	R primer (5′–3′)	CGCTGCCCAATCCCTTCA
AC008760.1	F primer (5′–3′)	TCCTGAGATGAAGCTGGAAATCAA
	R primer (5′–3′)	AGTTTCTACGGTGGAGGGGT
β-Actin	F primer (5′–3′)	AAACGTGCTGCTGACCGAG
	R primer (5′–3′)	TAGCACAGCCTGGATAGCAAC

F primer: forward primer; R primer: reverse primer

### Tube formation assay

100 μl ice‐cold Matrigel was added into a well of a 48‐wells plate for the HUVEC cells tube formation assay. 1 h later, 1 × 10^5^ HUVEC cells resuspended in 100 μl DMEM medium were added into the 48‐wells plate. After incubating at 37 °C in 5% CO_2_ incubator for 4 h, the tube formation status was photographed by light microscopy. The numbers of branch points were counted and analyzed by image J. 5 fields (200×) per chamber were observed for counting invaded cell numbers.

### Transwell assay

For the migration assay, 1 × 10^5^ HUVEC cells were suspended in 100 μl medium without FBS and seeded into an upper chamber (Corning, USA). Then, 800 μl complete DMEM medium containing 10% FBS was added to the lower chamber. For the invasion assay, Diluted Matrigel (1:5 dilution with the DMEM medium) was added into the cell culture inserts. Four hours later, 1 × 10^5^ HUVEC cells in 100 μl non-FBS DMEM medium were added into the upper chamber, while the lower chamber was filled with 800 μl of DMEM medium with 10% FBS. After culturing 24 h for migration test and 48 h for invasion test, the cells culture inserts were washed by PBS and stained with 4% paraformaldehyde. Then inserts were dyed by 0.1% crystal violet solution for 20 min. The dyed cells were photographed by light microscopy. 5 fields (200×) per chamber were observed for counting invaded cell numbers.

### Immunohistochemistry assay

Immunohistochemistry (IHC) analysis was performed as described previously [24]. IHC was performed using antibodies against CD31 (Cat# ab9498, Abcam, UK). Images were scanned using Pannoramic SCAN.

### BUC transcriptome data downloading and preprocessing

Transcriptome RNA-sequencing data of 414 BUC tumour tissues and 19 normal tissues were downloaded and extracted from The Cancer Genome Atlas (TCGA) data portal (https://portal.gdc.cancer.gov/). We excluded patients whose OS ≤ 30 days from this study because they might die of unpredictable factors such as hemorrhage and infection. The data utilized in the study were updated in March 21, 2021. Raw data of BUC patients were collected for further analyses. Transcriptome RNA-sequencing results and clinical data of BUC patients were combined into a matrix file by a merge script in the Perl language (http://www.perl.org/).

### Identification of angiogenesis-related long non-coding RNAs (ARLNRs) and the survival-related ARLNRs (sARLNRs)

Angiogenesis-related genes (ARGs) were extracted from The Molecular Signatures Database v4.0 (ANGIOGENESIS M14493 and WP_ANGIOGENESIS M39556, http://www.broadinstitute.org/gsea/msigdb/index.jsp). Then the angiogenetic scores of these ARGs were calculated according to their expression levels in BUC tissues. To further identify the ARLNRs, we conducted the Pearson correlation analysis to clarify the correlation between angiogenetic score and the expression of lncRNA in BUC tissue. A standard of |r| > 0.6 and *P* < 0.05 was used to screen the ARLNRs. Besides, we selected the sARLNRs by univariate COX analysis and survival packages of R software (*P* < 0.01). sARLNRs were further divided into deleterious and protective portions by the Hazard ratio (HR).

### Establish angiogenesis-related risk score model (ARRSM)

Through multivariate COX regression analysis, we established the ARRSM based on the selected sARLNRs. The score in the ARRSM was calculated based on the expression of sARLNRs together with the Cox regression coefficients. The formula was as followed, [Expression levels of KIRREL1-IT1 * (0.266525)] + [Expression levels of AC005625.1 * (− 0.135165)] + [Expression levels of AC018809.1 * (− 0.170812)] + [Expression levels of AC008760.1 * (− 0.133273)] + [Expression levels of AC083862.2 * (− 0.221852)]. BUC patients were separated into the high-risk group and the low-risk group according to the median score.

### Bioinformatics analysis

Receiver operating characteristic (ROC) curves were used to assess the sensitivity and specificity of the ARRSM and drawn by survival ROC package of R software. Gene set enrichment analysis (GSEA) was used to detect the different pathways of ARRSM. We evaluated the survival probabilities of patients in different risk groups by Kaplan–Meier survival curves. We conducted the univariate and multivariate Cox regression analyses to verify the independent prognostic factor of BUC. Nomograms were drawn to predict the survival probabilities of BUC patients by the rms package of R software.

### Statistical analysis

Statistical analysis was conducted by SPSS21.0 software (SPSS Inc, Chicago, IL) and GraphPad Prism8 (GraphPad Software Inc, La Jolla, CA). Data were expressed as means ± SD. The correlations between average expression of sARLNRs and clinicopathological characteristics of patients were evaluated using Fisher’s exact probability method. Student T-test, ANOVA and post-hoc test (Bonferroni method) were used for difference comparison of two or more groups. Pearson correlation analysis was utilized to analyze the correlation. *P* < 0.05 was considered a significantly statistical difference.

## Results

### Acquisition of sARLNRs

**Fig. 1:**
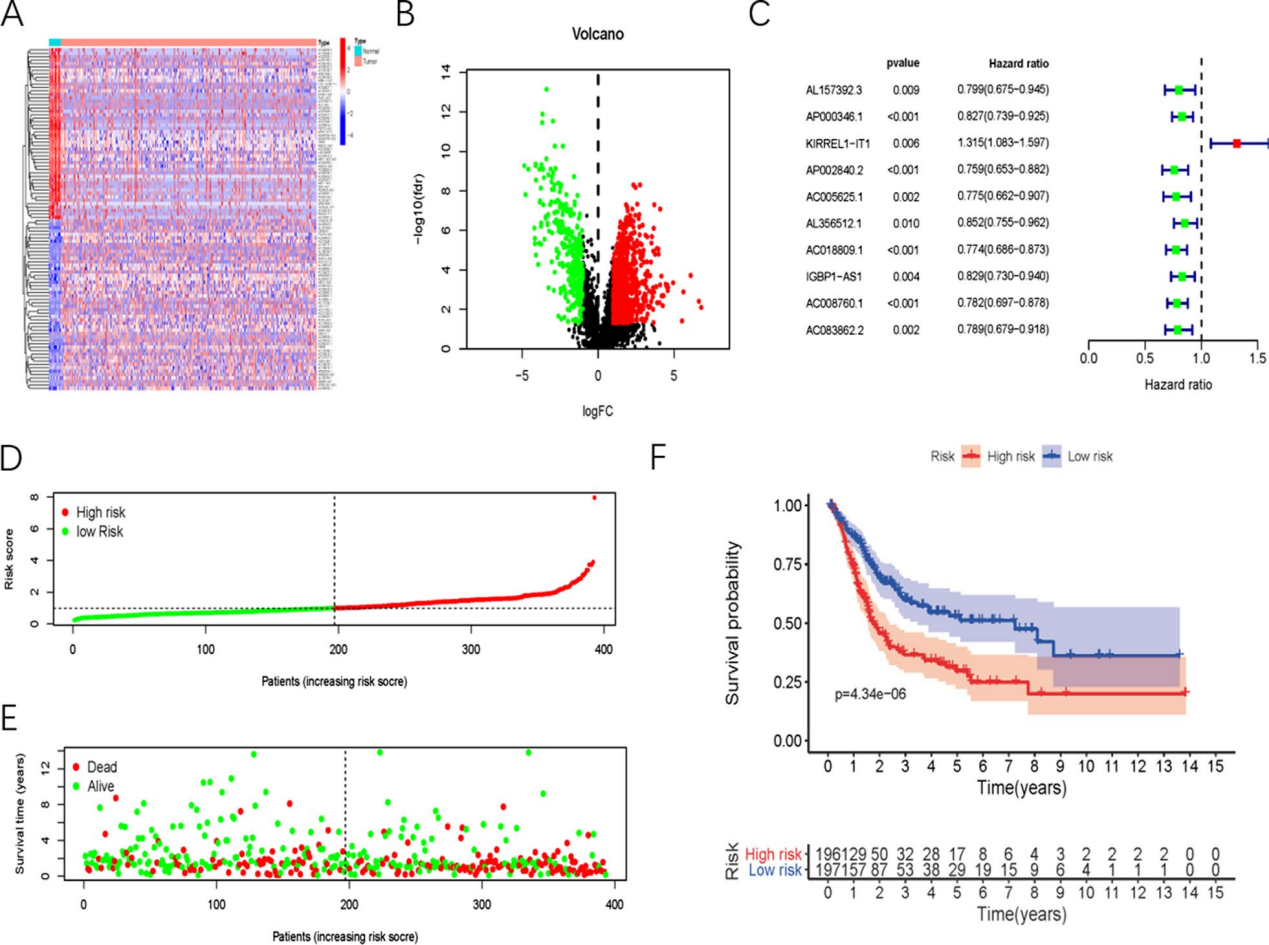
Differentially expressed survival-related ARLNRs in BUC samples and the construction of ARRSM. Heatmap (**A**) and volcano plot (**B**) illustrated the differentially expressed lncRNAs of tumor tissues and adjacent tissues of BUC patients in TCGA database. The red parts represent upregulated lncRNAs, and the blue parts represent downregulated lncRNAs in the heatmap. In the volcano plot, the green dots represent downregulated lncRNAs; the red dots represent the upregulated lncRNAs, and the black dots represent lncRNAs without differential expression. (log2 |FC| > 1 and P < 0.05). The hazard ratios of sARLNRs (sARLNRs: AL157392.3, AP000346.1, KIRREL1-IT1, AP002840.2, AC005625.1, AL356512.1, AC018809.1, IGBP1-AS1, AC008760.1 and AC083862.2) were demonstrated in the forest plot (**C**). The green parts represent protective sARLNRs, while the red parts represent deleterious sARLNRs. The risk-score distribution of the high-risk group and low-risk group (**D**). Survival status of patients in the high-risk group and low-risk group (**E**). Kaplan–Meier survival curve of the high-risk group and low-risk group (**F**)

Transcriptome RNA-sequencing data and clinical data of BUC patients were downloaded from TCGA database. Following that, lncRNAs data were extracted from the transcriptome data and 1282 differentially expressed lncRNAs were identified by limma algorithm, of which 310 lncRNAs were down-regulated and 972 lncRNAs were up-regulated (Fig. 1A, B). Then, we screened 72 ARGs in the ANGIOGENESIS M14493 and WP_ANGIOGENESIS M39556 of Molecular Signatures Database. After analyzing the correlations of the 1282 differentially expressed lncRNAs and 72 ARGs by Pearson correlation analysis, we found that 98 lncRNAs were associated with angiogenesis (|r| > 0.6 and *P* < 0.05). To better investigate the clinical significances of these ARLNRs, we then conducted the univariate COX regression analysis. Finally, 10 ARLNRs were identified to be relevant to OS of BUC patients (sARLNRs: AL157392.3, AP000346.1, KIRREL1-IT1, AP002840.2, AC005625.1, AL356512.1, AC018809.1, IGBP1-AS1, AC008760.1 and AC083862.2) (*P* < 0.05). As shown in the forest map, all sARLNRs but aside from KIRREL1-IT1 were regarded as protective factors (Fig. 1C).

### Establish the angiogenesis-related risk score model

To further validate the survival and prognostic relevance, we conducted multivariate COX regression analysis. Finally, KIRREL1-IT1, AC005625.1, AC018809.1, AC008760.1 and AC083862.2 were employed to establish the ARRSM (Table 3). Besides, the correlations of the five sARLNRs and OS were further illustrated via the survival curves in which the higher expression levels of AC005625.1, AC018809.1, AC008760.1 and AC083862.2 were correlated with the longer survival time, while the expression levels of KIRREL1-IT1 showed the opposite conclusion (Additional file 1: Fig. S1). According to the median risk score, BUC patients were divided into the high-risk group and the low-risk group (Fig. 1D). With the increasing risk score, the mortality rate of BUC patients and the expression levels of KIRREL1-IT1 constantly increased, while AC005625.1, AC018809.1, AC008760.1 and AC083862.2 expressed decreasingly (Fig. 1E). Kaplan–Meier analysis illuminated that BUC patients in the low-risk group exhibited a longer survival time than those in the high-risk group (Fig. 1F).

**Table 3:** The results of multivariate Cox regression analysis

Gene	Coefficients	HR	HR.95% high	HR.95% low	P value
KIRREL1-IT1	0.26652	1.30542	1.58871	1.07264	0.00782
AC005625.1	− 0.13517	0.87357	1.03632	0.73638	0.1209
AC018809.1	− 0.17081	0.84297	0.96365	0.73741	0.01233
AC008760.1	− 0.13327	0.87522	0.99154	0.77254	0.03632
AC083862.2	− 0.22185	0.80103	0.93114	0.68911	0.00386

*HR* hazard ratio

### The relationship of ARRSM and clinicopathological feature

**Fig. 2:**
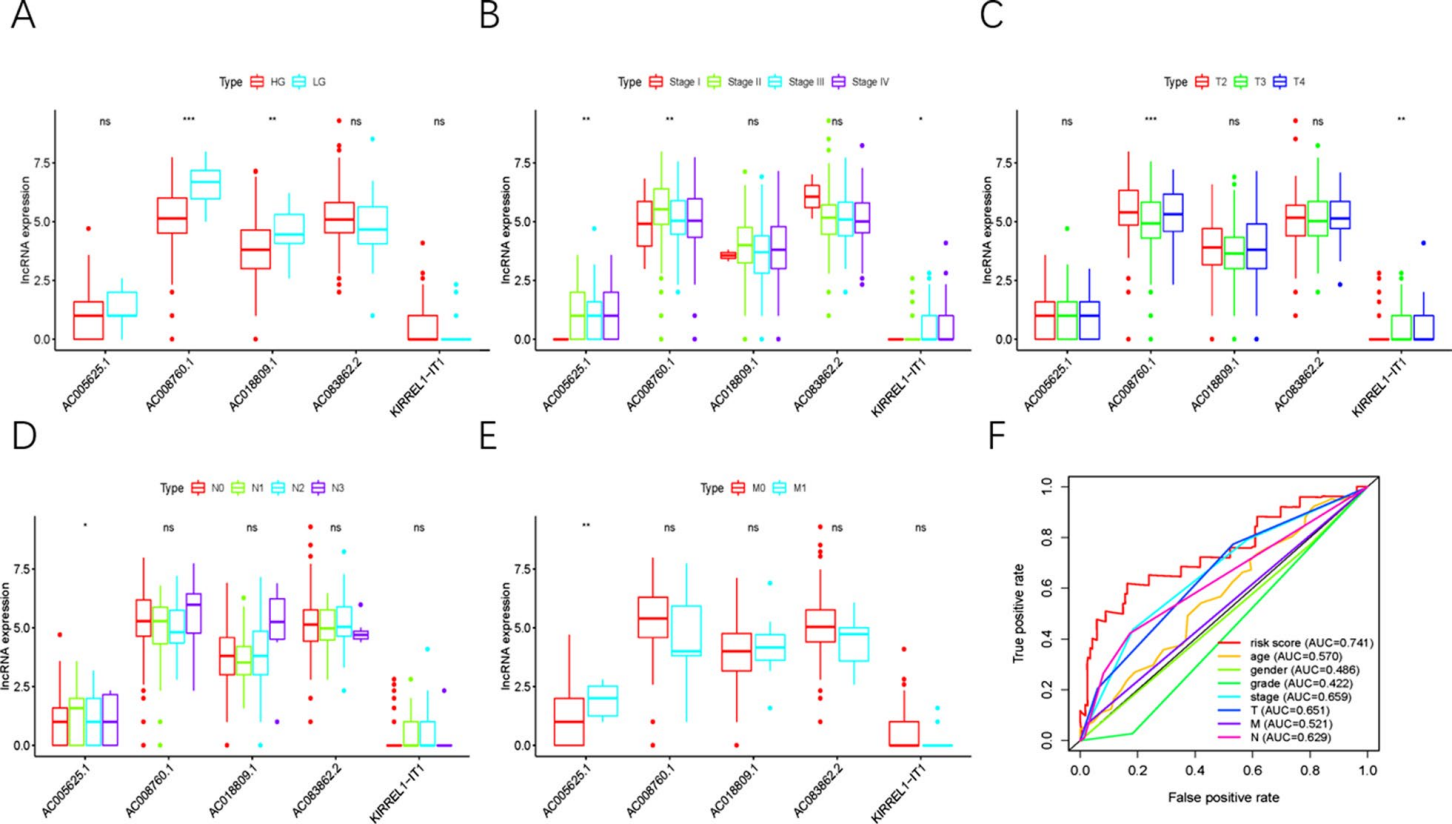
The relationship of ARRSM and clinicopathologic features and the ROC curve of ARRSM. AC018809.1 and AC008760.1 expressed more in patients with the advanced stage (**A**). The expression levels of AC005625.1 and AC008760.1 were enhanced in the early stage but KIRREL1-IT1 increased (**B**). KIRREL1-IT1 expressed more in the advanced T-stage while AC008760.1 dropped (**C**). The expression levels of AC005625.1 decreased in the advanced N-stage but increased in advanced M-stage (**D**, **E**). (***P < 0.001; **P < 0.01; *P < 0.05; ns: *P* > 0.05) The areas under curves (AUCs) were evaluated by ROC curves. The AUCs’ values of risk score, age, gender, grade, stage, T-stage, M-stage and N-stage were 0.741, 0.57, 0.486, 0.422, 0.659, 0.651, 0.521 and 0.629 respectively (**F**)

In order to explore the relevance of sARLNRs and clinical features of BUC patients, we used ggpubr package of R language to analyze the correlations of ARRSM and the clinical characteristics, such as grade, stage, TNM stage. We found the expression levels of AC018809.1 and AC008760.1 decreased in patients with advanced grade (Fig. 2A). The expression levels of AC005625.1 and AC008760.1 increased in the more advanced stage while KIRREL1-IT1 dropped (Fig. 2B). Besides, the expression level of KIRREL1-IT1 was elevated in the advanced T-stage but AC008760.1 decreased (Fig. 2C). AC005625.1 expressed more in the advanced N-stage and M-stage (Fig. 2D, E).

### The clinical application of ARRSM and gene set enrichment analysis (GESA)

**Fig. 3:**
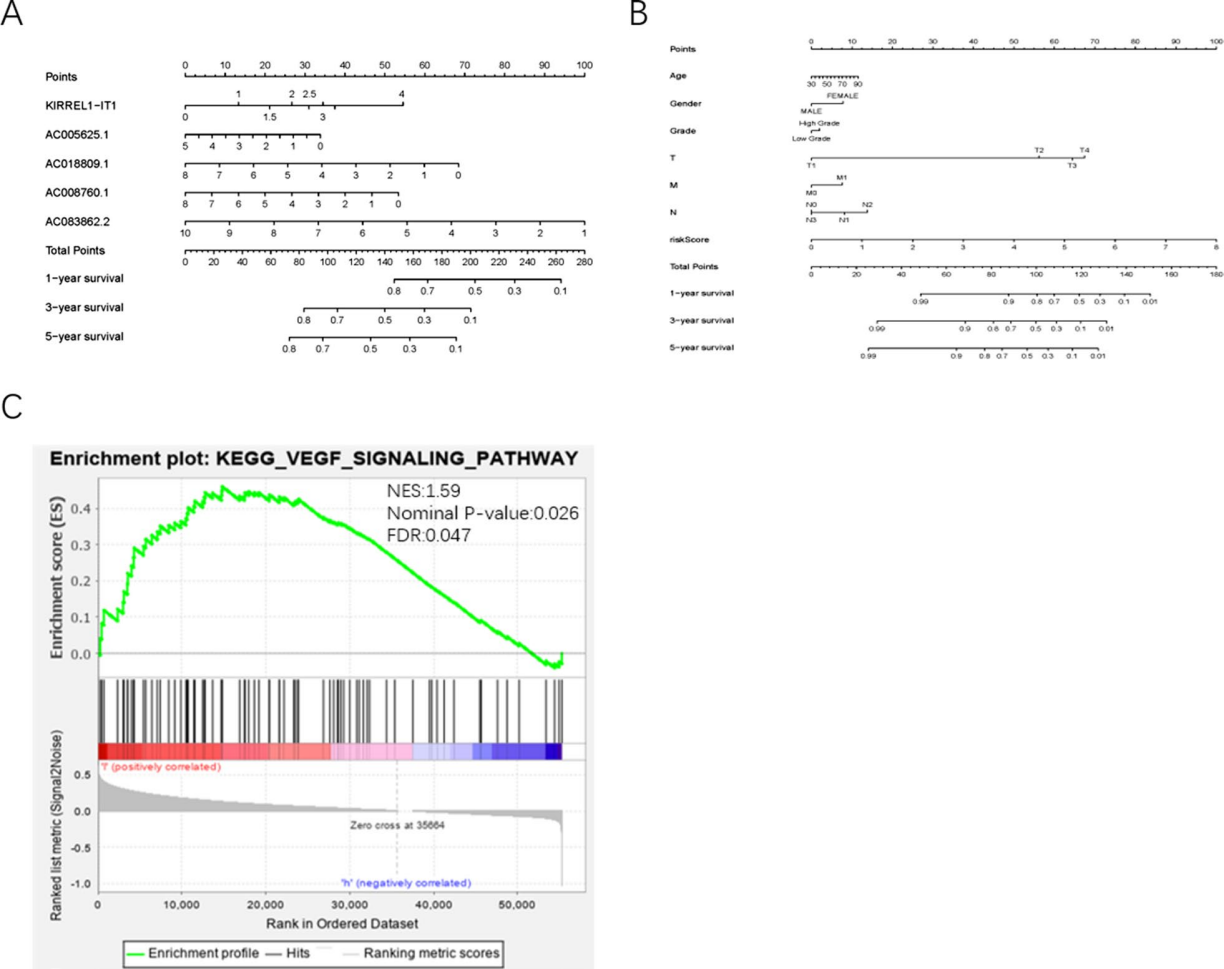
Nomograms and GSEA of ARRSM. Nomogram (**A**) was used to predict 1-, 3-, and 5-year survival probability of BUC patients by evaluating the expression levels of five sARLNRs. The clinical features and risk score were utilized to predict the 1-, 3-, and 5-year prognosis of BUC patients (**B**). GSEA results implied significant enrichment of the VEGF pathway in the high‐risk group (**C**)

Next, we performed univariate analysis to analyze the independent risk factor of BUC patients, and the results showed gender, stage, T-stage, N-stage, M-stage and risk score were significantly correlated to prognosis (*P* < 0.05). However, through the multivariate analysis, only risk score could be served as an independent risk factor of BUC patients (Table 4). Next, we calculated the areas under curves (AUCs) for receiver operating characteristic (ROC) curves of ARRSM and clinical characters, and found the AUCs of risk score, age, gender, grade, stage, T-stage, M-stage and N-stage were 0.741, 0.57, 0.486, 0.422, 0.659, 0.651, 0.521 and 0.629 respectively (Fig. 2F). The highest AUC was risk score, which meant the ARRSM was the most accuracy independent risk factor of BUC patients. We normalized the points of each BUC patient to a distribution ranging from 0 to 100, and then we calculated the 1-year, 3-year and 5-year survival probabilities of BUC patients by drawing a vertical line (clinical features and the expression levels of sARLNRs) between the total points axis and each prognosis axis (Fig. 3A, B). The nomograms will provide a novel diagnostic method for clinical workers to assess the prognosis of BUC patients. These results illustrated that the ARRSM could be served as a promising risk score model in predicting the prognosis of BUC patients.

**Table 4:** Univariate and multivariate COX analysis of UBC patients

Variables	Univariate analysis				Multivariate analysis			
	HR	HR 95% low	HR 95% high	P value	HR	HR 95% low	HR 95% high	P value
Age	1.023152	0.997282	1.049694	0.079829	1.012561	0.985234	1.040647	0.371155
Gender	0.592245	0.353164	0.993178	0.047042	0.625978	0.364538	1.074915	0.089491
Grade	0.270351	0.037141	1.967895	0.196517	0.867941	0.109308	6.891722	0.893421
Stage	1.804371	1.290509	2.522841	0.000557	1.187281	0.595591	2.366786	0.625752
T-stage	1.684872	1.173265	2.419568	0.004722	1.359828	0.826785	2.236534	0.226006
M-stage	2.520036	1.005311	6.317033	0.048694	1.456805	0.526254	4.032801	0.468921
N-stage	1.556737	1.207657	2.006723	0.000634	1.280255	0.797118	2.056222	0.306788
Risk score	2.043276	1.573784	2.652826	8.12e−08	2.054913	1.558068	2.710195	3.39e−07

*HR* hazard ratio

Then, we used kyoto encyclopedia of genes and genomes (KEGG) pathway analysis of GSEA to further investigate the underlying mechanisms of ARRSM. We found that the high-risk group was closely related to the VEGF signaling pathway (Fig. 3C). These results motivated us to further discover the underlying mechanisms of angiogenesis and immune cell infiltration in the future studies.

### AC005625.1 and AC008760.1 expressed lowly in BUC cell lines and tumour tissues, especially in patients with more advanced T stages

**Fig. 4:**
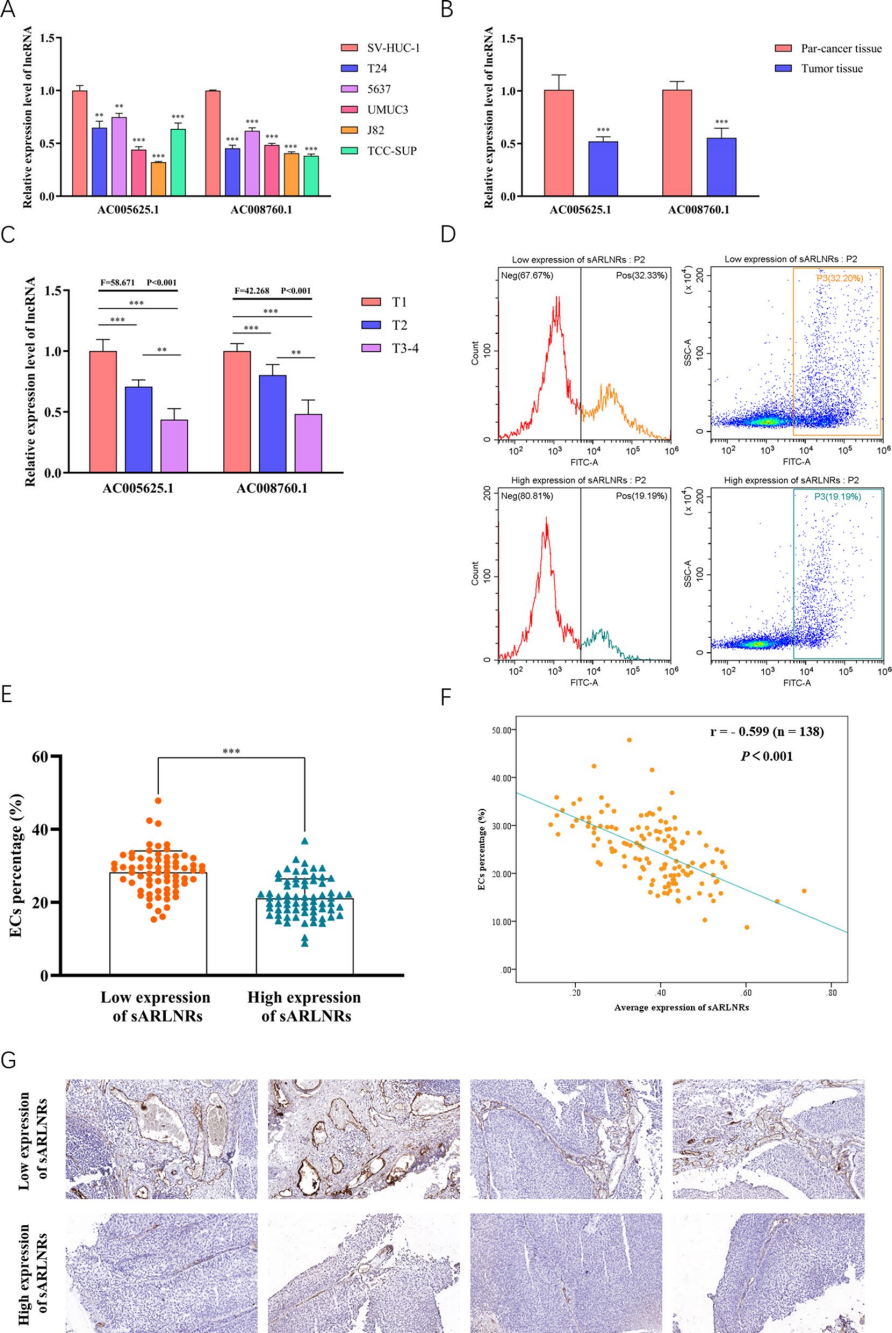
The expression levels of AC005625.1 and AC008760.1 in cell lines and clinical samples. The qPCR results of the expression levels of AC005625.1 and AC008760.1 in BUC cell lines (T24, 5637, UM-UC-3, 5637, J82 and TCC-SUP) and urothelial cells (SV-HUC-1) (n = 3) (**A**), BUC tumour tissues and adjacent tissues (n = 138) (**B**, **C**). The percentage of ECs in BUC tumour tissues with low (top) and high (down) average expression of AC005625.1 and AC008760.1 (**D**). The quantitative analysis of ECs percentage in BUC tumour tissues (n = 138) (**E**). The Pearson correlation analysis of ECs percentage and average expression of AC005625.1 and AC008760.1 (n = 138, Pearson's correlation coefficient = − 0.599, *P* < 0.001) (**F**). IHC was used to determine the EC (identified by CD31 positivity) percentage in tumour tissues of 138 BUC patients (Scale bar: 50 μm) (***P < 0.001, **P < 0.01 and *P < 0.05 represent significant differences between the two groups or compared to Control group)

In order to verify the expression levels of sARLNRs in various BUC cell lines and clinical samples, we detected the expression levels of AC005625.1 and AC008760.1 in SV-HUC-1, different BUC cell lines, tumour tissues and adjacent normal tissues. Compared with SV-HUC-1, the expression levels of AC005625.1 and AC008760.1 were significantly lower in T24, 5637, UMUC-3, J82 and TCC-SUP BUC cell lines (Fig. 4A). Besides, we found that AC005625.1 and AC008760.1 expressed lower in tumour tissues than those in adjacent normal tissues (Fig. 4B), with the lower expression in more advanced T stage (Fig. 4C).

### AC005625.1 and AC008760.1 expression was negatively related to ECs percentage, tumour size and muscle invasion status

To further investigate the clinical significance of the sARLNRs, we first divided the 138 BUC patients into sARLNRs-high-expression (n = 69) and sARLNRs-low-expression (n = 69) groups according to the average expression of AC005625.1 and AC008760.1 (the cut-off value: 0.4057). As illustrated in Table 1, the expression of AC005625.1 and AC008760.1 was significantly related to ECs percentage, tumour size and muscle invasion status, while there was no remarkable correlation with gender, age, distant metastasis, lymph node status, or nidus number. To further discover the correlation of the sARLNRs and tumour-related angiogenesis, we detected the percentage of EC subtype in tumour tissues by FACS. As illustrated in Fig. 4D, E, a prominently higher proportion of EC was observed in tumours with lower average expression of AC005625.1 and AC008760.1. The result of Pearson correlation analysis suggested the significant negative correlation between sARLNRs expression and ECs percentage (r = − 0.599, *P* < 0.001) (Fig. 4F). Furthermore, the higher percentage of EC in tumour tissues with lower expression of AC005625.1 and AC008760.1 was also validated by IHC (Fig. 4G).

### Knockdown of AC005625.1 and AC008760.1 in BUC cells promoted tube formation, migration and invasion of HUVECs

**Fig. 5:**
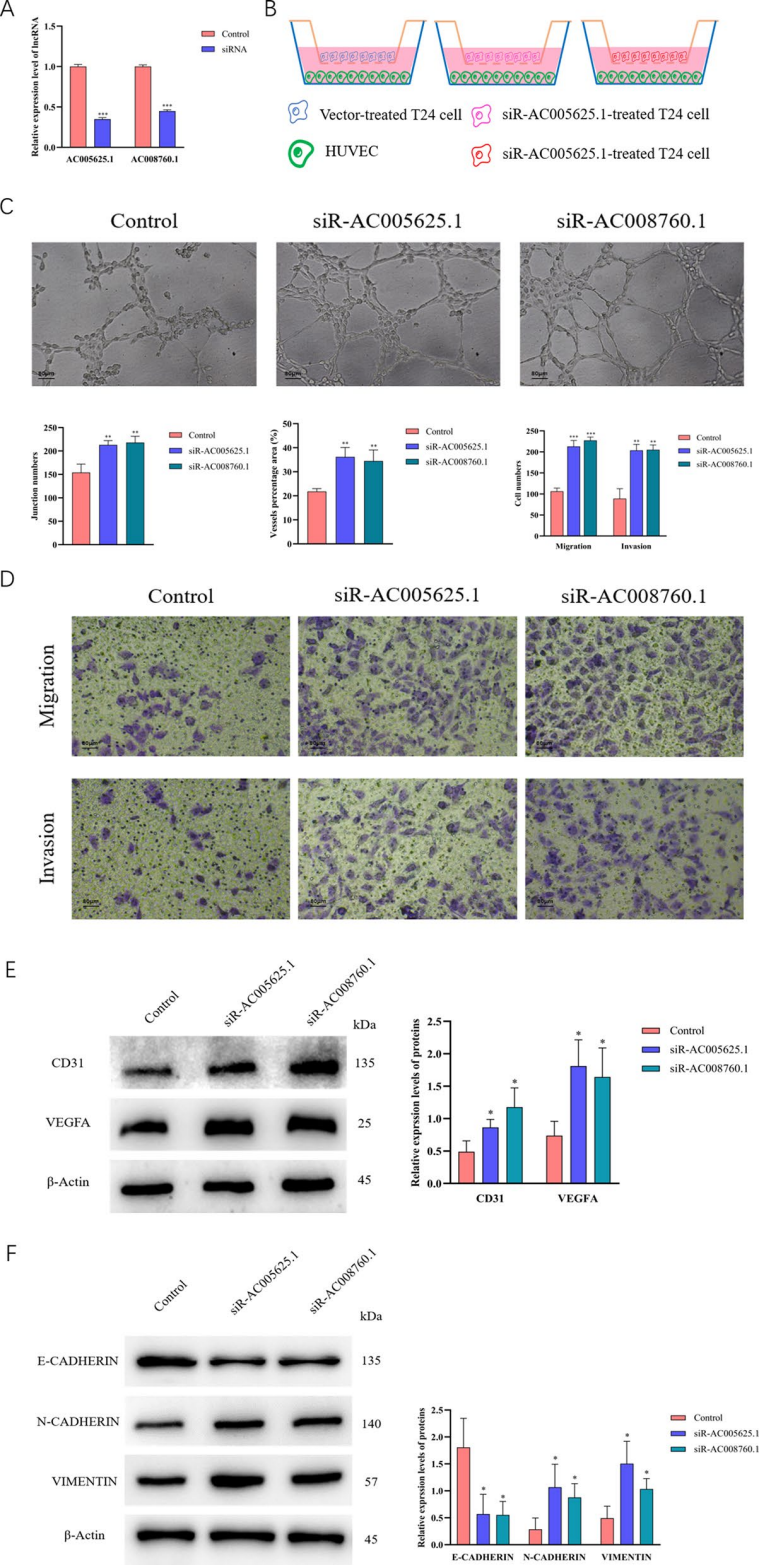
The effects of AC005625.1 and AC008760.1 on tube formation, invasion and migration. SiR-AC005625.1 and siR-AC008760.1 significantly attenuated the expression of AC005625.1 and AC008760.1 in T24 cells, respectively (n = 3) (**A**). Co-culture models of HUVECs and different T24 cells (**B**). Knockdown of AC005625.1 and AC008760.1 in T24 cells elevated the tube formation ability of co-cultured HUVECs (top) and quantitative analysis (down) (n = 3, Scale bar: 80 μm) (**C**). The migration and invasion abilities of HUVEC cells (down) and quantitative analysis (top) (n = 3, Scale bar: 80 μm) (**D**). CD31, VEGF-A, VIMENTIN and N-CADHERIN expressed more in HUVECs co-cultured with siR-AC005625.1 and siR-AC008760.1-treated T24 cells, but ECADHERIN changed reversely (left) and quantitative analysis (right) (n = 3) (**E**, **F**). (***P < 0.001, **P < 0.01 and *P < 0.05 represent significant differences compared to Control group)

To further determine the effects of AC005625.1 and AC008760.1 in tumour-related angiogenesis, we knocked down the AC005625.1 and AC008760.1 using siRNAs in T24 cells (Fig. 5A) followed by the co-culture of T24 cells and HUVEC cells to simulate tumour microenvironment of BUC (Fig. 5B). Next, we performed HUVEC cells tube formation and Transwell experiments to explore the angiogenesis-related roles of AC005625.1 and AC008760.1 in tumour microenvironment of BUC. As shown in Fig. 5C, D, the tube formation, migration and invasion abilities of HUVEC cells co-cultured with siRNAs-treated T24 cells were prominently enhanced. Then, we found that the expression of angiogenesis-related proteins (CD31 and VEGF-A) (Fig. 5E) and the key proteins in the epithelial–mesenchymal transition pathway (VIMENTIN and NCADHERIN) (Fig. 5F) increased in HUVECs which were co-cultured with siR-AC005625.1 and siR-AC008760.1-treated T24 cells, while E-CADHERIN decreased. These results demonstrated that knockdown of AC005625.1 and AC008760.1 in BUC cells promoted tumour-related angiogenesis by augmenting the tube formation, invasion and migration abilities of HUVECs in the TME.

## Discussion

Increasing evidence has unraveled that angiogenesis is crucial for tumour progression and is highly dependent on VEGF expressed by most malignant tumours [25–27]. Additionally, FRS2-mediated signals were validated to promote tumour angiogenesis and predict poor prognosis in prostate carcinoma, high-grade serous ovarian cancer and liposarcoma [28–30]. The finding of recurrent ADGRG6 enhancer mutations also facilitated our knowledge of underlying molecular mechanisms of pathological angiogenesis in the highly vascularized cancers [31]. Hypoxia-inducible factor 1 (HIF1) as a heterodimeric transcription factor composed of HIF1α and HIF1β subunits, is also regarded as a major angiogenetic regulator in the tumour microenvironment [32]. Recently, a phase II trial illuminated that ramucirumab (a VEGF receptor‑2 antibody) combined with second-line docetaxel, seemed to show the beneficial outcomes [33]. However, anti-angiogenesis has not yet been verified as a therapy strategy with higher priority in urothelial carcinoma. Besides, much less is known about the potential roles of angiogenesis-related biomarkers.

Recent studies have demonstrated that lncRNAs regulated the various processes involved in angiogenesis directly or indirectly by targeting different angiogenetic molecules [34]. Because of the influential roles, lncRNAs are rapidly emerging as a type of promising drug target and candidate. Lin et al. reported that lncRNA UBE2CP3 augmented hepatocellular carcinoma cell secretion of VEGFA and promoted angiogenesis by activating ERK1/2/HIF1α/VEGFA axis [35]. LncRNA PAXIP1-AS1 was identified to boost migration and angiogenesis of glioma by upregulating ETS1-midiated KIF14 expression [36]. Aside from the pro-angiogenetic potential, Chang et al. found lncRNA LINC00320 suppressed tumourigenicity of glioma and angiogenesis through reduction of NFKB1-regulated AQP9 [37]. A growing body of ARlncRs not only provided therapeutic targets, but were also promising for diagnosis and prognosis evaluation. For example, lncRNA PANTR1 was related to poor prognosis and promoted angiogenesis and apoptosis in clear cell renal cell cancer [38].

In this study, we identified five ARlncRs with prominent clinical significance of BUC. Following the establishment of ARRSM based on the five ARlncRs, BUC patients could be divided into the high-risk group and the low-risk group by scores reflecting the different OS. Besides, through the in vitro experiments, we not only found the lower expression of AC005625.1 and AC008760.1 in BUC cells and tumour tissues, but also validated the negative correlation with T stage, tumour size and muscle invasion status. More importantly, we found that AC005625.1 and AC008760.1 expression was negatively related to ECs percentage in tumour tissues. Besides, the angiogenesis-inhibited roles and underlying mechanisms of AC005625.1 and AC008760.1 were further validated in the co-culture models of HUVEC cells and T24 cells.

Identified as an autophagy-related lncRNA, AC008760.1 was demonstrated to be potential for predicting poor prognosis of colorectal cancer [39], but few studies reported the roles of KIRREL1-IT1, AC005625.1, AC018809.1, AC008760.1 and AC083862.2 in the realm of angiogenesis. It is clear that tumour vascularization, as a hallmark feature of cancer, is not a just innocent bystander, but in many cases regulate crucial process of tumour progression. Therefore, identifying more promising angiogenetic targets merit further attention. Here, we established an ARRSM based on five sARLNRs, which not only provided more therapeutic targets, but also better evaluate prognosis of BUC patients from the perspective of tumour vascularization.

Although we elucidated the potential of ARRSM for prognosis assessment of BUC patients and validated clinical significances and angiogenetic correlation of sARLNRs through a series of in vitro experiments based on cell lines and clinical samples, some limitations remain to be further strengthened in future study. First, proteomics and metabonomics assays should be implemented to reveal wider and deeper perspectives on tumour angiogenesis. Next, the more underlying mechanisms of these sARLNRs of ARRSM should be explored through a chain of in vivo and in vitro experiments in subsequent study.

## Conclusion

In the present manuscript, we unraveled the potential of sARLNRs for prognosis evaluation of BUC from the angiogenetic perspective and ascertained their clinical significance. These findings not only help to establish a link between lncRNA and tumour angiogenesis, but also provide a reliable and accurate ARRSM developed based on some novel targets for future antiangiogenic therapies.

## Supplementary Information

**Additional file 1: Figure S1.** Survival curves of five sARLNRs.

## Data Availability

Authors can provide all of datasets analyzed during the study on reasonable request.

## Abbreviations

ARLNRs: Angiogenesis-related long non-coding RNAs
ARRSM: Angiogenesis-related risk score model
BUC: Bladder urothelial carcinoma
FBS: Fetal bovine serum
GSEA: Gene set enrichment analysis
IME: Immune microenvironment
MIBC: Muscle-invasive bladder cancer
NMIBC: Non-muscle-invasive bladder cancer
OS: Overall survival
QOL: Quality of life
sARLNRs: Survival-related ARLNRs
lncRNAs: Long non-coding RNAs
VEGF: Vascular endothelial growth factor
