# Supplementary figures and images for: A novel risk score model based on five angiogenesis-related long non-coding RNAs for bladder urothelial carcinoma
Source: Cancer Cell Int. 2022 Apr 19;22:157. doi: 10.1186/s12935-022-02575-1 (PMC9019982; doi:10.1186/s12935-022-02575-1)

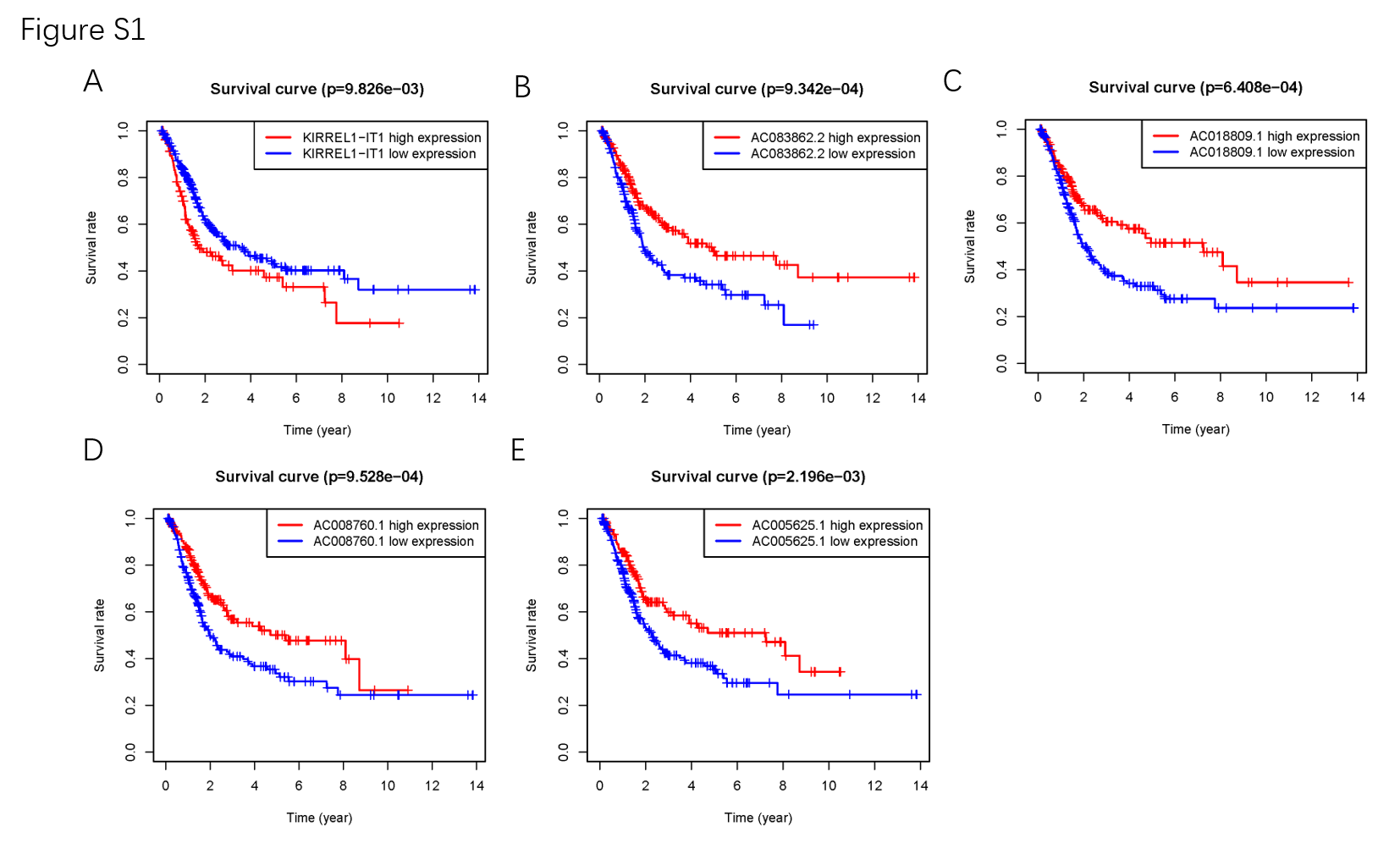

Supplement: Supplementary file 1 — Additional file 1: Figure S1. Survival curves of five sARLNRs. [file 12935_2022_2575_MOESM1_ESM.tif]
